# The scale-up and integration of contraceptive service delivery into nursing school training in the Democratic Republic of the Congo

**DOI:** 10.1093/heapol/czab014

**Published:** 2021-05-02

**Authors:** Alexis Ntabona, Arsene Binanga, Mr Désiré Josué Bapitani, Beatrice Bobo, Bitshi Mukengeshayi, Pierre Akilimali, Gloria Kalong, Zenon Mujani, Julie Hernandez, Jane T Bertrand

**Affiliations:** Consultant, ExpandNet; Tulane International LLC, Kinshasa, DRC; D6 Directorate in Charge of Nursing Schools, Ministry of Health; D6 Directorate in Charge of Nursing Schools, Ministry of Health; Action Santé et Développement (ASD); Kinshasa School of Public Health; Tulane School of Public Health and Tropical Medicine (SPHTM), 1440 Canal St, Suite 1900, New Orleans, LA 70112, USA; Tulane International LLC, Kinshasa, DRC; National Programme for Reproductive Health (PNSR), Maternité de Kintambo, Kinshasa, DRC; Tulane School of Public Health and Tropical Medicine (SPHTM), 1440 Canal St, Suite 1900, New Orleans, LA 70112, USA; Tulane School of Public Health and Tropical Medicine (SPHTM), 1440 Canal St, Suite 1900, New Orleans, LA 70112, USA

**Keywords:** Scale-up, nursing students, family planning, contraception, community-based services, Democratic Republic of the Congo (DRC)

## Abstract

In Kinshasa, Democratic Republic of the Congo (DRC), modern contraceptive prevalence is low by international standards: 29.6% as of 2020. A 2015 pilot study demonstrated the feasibility and acceptability of using medical and nursing students to administer DMPA-SC (the subcutaneous injection) among other methods at the community level. The more far-reaching discovery was the potential of clinically trained students to increase access to low-cost contraception in the short-run, while improving the quality of service delivery for future generations of healthcare providers. Scale-up involved integrating the family planning curriculum into the training of nursing students, including classroom instruction in contraceptive technology and service delivery, coupled with a year-long field practicum in which students offered a range of contraceptive methods during intermittent outreach events, door-to-door distribution or sales from their homes. Starting in 2015, a multi-agency team consisting of an international non-governmental organizations (NGO), several Ministry of Health directorates and a local NGO used the ExpandNet/WHO framework to guide this scale-up. This article details the nine steps in the scale-up process. It presents results on increases in contraceptive uptake, feedback from participating nursing school personnel and the employment experience of the graduates from this programme. Between 2015 and 2019, the family planning curriculum was incorporated into 30.8% of the 477 nursing schools in 7 of the 26 provinces in the DRC. Students delivered 461 769 couple-years of protection (the key output indicator for family planning programmes). Nursing school personnel were strongly favourable to the approach, although they needed continued support to adequately implement a set of additional interventions related to the service delivery components of the new training approach. Post-graduation, only 40.1% of graduates had paid employment (reflecting the staggering unemployment in the DRC); among those, over 90% used their family planning training in their work. We describe the multiple challenges faced during the scale-up process and in planning for expansion to additional schools.

**KEY MESSAGES**

- Scaling up the competency-based training on family planning community-based services into the curriculum of nursing students in the Democratic Republic of the Congo has expanded contraceptive access and strengthened the capacity of the future generation of this category of healthcare providers.
- The primary challenge to scale-up of this innovative approach is the cost of the community based service delivery of contraceptives.

## Introduction

The Democratic Republic of the Congo (DRC) has the fourth largest population in sub-Saharan Africa and is one of the fastest-growing countries in the world. The modern contraceptive prevalence rate among married women (MCPR) rose from 7.8% (MPSMRM *et al.*, 2013–14) to 17.6% (INS, 2019), but is still low by international standards. For the capital city, Kinshasa, the MCPR was 29.6% in 2020 (PMA, 2020). A recent article by Kwete *et al.* (2018) outlines the promising momentum for family planning: political support, increased donor investment, climate for innovation and a cohesive mechanism that unifies family planning stakeholders. The article also identifies formidable challenges: political uncertainty (prior to the last presidential election), logistics and management of service delivery, contraceptive supply chain management, high fertility norms and cost barriers. To this list, one must also add the CoVid-19 crisis, which threatens all aspects of an already fragile health system. In the past decade, the DRC government has shown its support for family planning, especially as an element in its pursuit to become an emerging nation by 2030. Numerous donors and international/national non-governmental organizations (NGO) support the government efforts to increase access to quality family planning services in this vast country.

The government and its partners have tested different strategies to increase contraceptive uptake in both urban and rural settings, with varying degrees of success. In 2015, a pilot test conducted in Kinshasa yielded promising results of a new approach: to train medical and nursing students in family planning counselling and service delivery, emphasizing provision at the community level (Binanga and Bertrand, 2016; Bertrand *et al.*, 2017). The innovative aspect of the pilot was having these students administer the subcutaneous injection, DMPA-SC (in addition to pills, condoms and CycleBeads) under the supervision of their instructors, given that at the time only certified doctors and nurses administered injections.

The results of the pilot demonstrated not only acceptability and feasibility, but revealed a more far-reaching finding: the untapped potential of these students as community-level providers of contraception. Even before results dissemination, local family planning stakeholders began to talk about replication and scale-up, which would be limited to nursing schools only. (Medical schools operate more autonomously in the DRC, making standardization of the curriculum difficult.) Fortuitously, programme managers involved in the pilot knew of the ExpandNet/WHO framework and enlisted assistance from its project staff to guide the process of institutionalizing the family planning curriculum into the nursing schools (WHO and ExpandNet, 2010, 2011).

The prospect of scale-up (referred to locally as ‘institutionalization’ in the context of this work) met with enthusiasm from several key actors. For the D6 (the Directorate of the Ministry of Health (MOH) responsible for the 477 nursing schools in the country), it represented an opportunity to reinforce the curriculum with updated information and practical training in an area of maternal and child health. It introduced the approach of competency-based training. And it provided its graduates with much-needed skills in client–patient interaction, counselling and contraceptive provision.

Family planning programme managers saw multiple benefits of scale-up. In the short term, it offered a means of providing contraceptive services in an expanded number of underserved communities in Kinshasa and subsequently in the adjacent province of Kongo Central. It brought services closer to potential clients, who often experienced barriers of cost and long waiting times in fixed facilities in their neighbourhoods. Methods could be provided at lower cost or free on certain campaign days. And the training promised to increase service quality. In the long term, the scale-up of this module would ensure a trained, experienced cadre of health personnel for contraceptive delivery for future generations.

In short, scale-up had the dual objective of strengthening the capacity of DRC nursing schools to provide training in family planning, while also expanding access to contraceptive services delivered at the community level.

This article documents the scale-up process that has taken place since 2016, guided by the ExpandNet/WHO framework, a tested approach to scale-up of successfully piloted interventions in public health (WHO and ExpandNet, 2010, 2011). We begin by describing the process in terms of the nine steps of the strategy, which has led to incorporation of an adapted community-based family planning module into the curriculum of 147 nursing schools in 7 of the 26 provinces of the DRC. We then present the methodology used for the research conducted on this process.

The aim of this article is to present evidence on three research questions:

1. How did the project contribute to expanding access and increasing contraceptive uptake in the DRC?
2. How did key personnel in the participating D6 schools react to this change in the nursing school curriculum?
3. What has been the experience of students post-graduation, especially in terms of using their training to deliver services?

## The process of scale-up, based on the ExpandNet/WHO framework

ExpandNet is an informal network of global health and development professionals who seek to advance the science and practice of scale-up. This group developed a nine-step framework to systematically analyse and support the implementation of selected necessary actions for sustainable scale-up. Guiding principles include systems thinking, a focus on sustainability, enhancing scalability and respect for human rights, equity and gender perspectives. Their tools include Practical guidance for scaling up health service innovations, Beginning with the end in mind: Planning pilot projects and other programmatic research for successful scaling up, and Nine steps for developing scaling-up strategy (WHO and ExpandNet, 2010, 2011).

In the case of the DRC, the early enthusiasm for the approach of using nursing students to deliver contraceptive services prompted interest in scale-up. The aspirational objective is to make this skills-based training a permanent part of the curriculum and reach all nursing schools nationwide, albeit in staggered fashion.

### Step 1: Clarify what is the innovation and its scalability potential

The basic nursing training in the DRC occurs via a network of schools known as ITM (Institut des Techniques Médicales), which constitute a specialized track of secondary school. Students wishing to take this option must have completed primary school through the equivalent of eighth grade (in the US system) or the ‘troisième’ (third level) in the Belgian/French system, and be able to pay school fees. Students generally range in age from 15–18. The innovation consisted of introducing a competency-based training approach for third year (and subsequently fourth year) nursing students in contraceptive counselling and service delivery, which in itself is not necessarily innovative. However, the theoretical training was accompanied with an extended field practicum in which the students regularly served as community-based distributors in communities near their schools for the reminder of the academic year. Specifically, they counselled interested clients in contraception methods, advantages of family planning, side effects and their management, and related topics. In addition, they provided (initially four, later six) contraceptive methods to interested clients at the community level, under the supervision of their instructors, either by going door-to-door or receiving interested clients at an outreach event (‘campaign’) in the community. The methods were Implanon NXT, DMPA-SC, CycleBeads, oral pills, condoms and emergency contraception. The curriculum previously designed to train community health workers in family planning was adapted for this purpose. The nursing school model tested in Kinshasa was replicated in Kongo Central in 2016. The scale-up to additional provinces began with the cohort of third-year nursing students in 2017–18.

### Step 2a: Identify the organizations that seek or are expected to adopt and implement the innovation

The initial idea for the 2015 pilot study came from Tulane International LLC (TILLC), an international non-governmental organization (iNGO) that manages several research, advocacy and service delivery projects in Kinshasa. At the time, medical norms in the DRC dictated that only doctors and nurses could give injections. One notable exception was that medical and nursing students were also allowed to do so under the supervision of their instructor(s). Around this time, DMPA-SC (the contraceptive injection administered with a short needle) had recently become available and had proven popular in other sub-Saharan African countries. In 2015, TILLC worked with the D6 (the user organization), the National Programmes for Reproductive Health (PNSR) and for Adolescent Health (PNSA), and a local NGO, Action Santé et Développement (ASD), to organize the pilot test. The local NGO identified and recruited 10 nursing schools from the D6 network in Kinshasa to participate in the pilot, worked with the schools to introduce family planning into the training of third year nursing students, and assisted in setting up outreach events in nearby health zones at which the students gave counselling and delivered family planning services. Data were collected from clients at the time of the initial injection and again at 6 months regarding the experience, and from providers also at 6 months. The key results were that the clients had had positive experiences with the method and were satisfied with the services received by these providers (Bertrand *et al.*, 2017).

In early 2015, the Director of the D10 presided over a meeting of family planning stakeholders to review the design of the pilot study and ensure buy-in from relevant parties. Most of the same governmental authorities were present at the dissemination of findings in December 2015.

The iNGO subsequently sought technical assistance from the ExpandNet team and then assumed the role of convener in engaging three Directorates within the MOH in the scale-up process^1^:

1. D10, the Directorate responsible for reproductive health and safe motherhood, represented by the PNSR and the PNSA;
2. D6, the Directorate that oversees the 477 teaching institutions for nurses^2^ nationwide;
3. D5,^3^ the Directorate that manages the National Health Information System (SNIS, Système National d’Information Sanitaire) that compiles all service statistics at the health facility and community level.

In sum, the impetus for the scale-up process (i.e. institutionalization and expansion) resulted from the collective efforts of the user organization (the D6), which had embraced this innovation as a means of strengthening training in its network of schools, and the multiple collaborating organizations described above.

### Step 2b: Identify which user organization should lead the scaling-up process

All the stakeholders agreed that the D6—responsible for nursing training nationwide—should take the lead for the first stage of the scaling-up process, which focused on streamlining the innovation into the network of nursing schools. Thus, the D6 became the user organization.

The DRC is divided in 519 health zones, each with a Chief Medical Officer and other health personnel responsible for primary healthcare delivery. The D6 nursing schools operate within this system; thus, coordination was essential between the nursing schools and health zone authorities. In addition, the D10—which provides oversight to the PNSR—was particularly critical, since the MOH would need to authorize and support this new approach of using nursing students to provide community-based family planning services. The D6 at the national level looked to the lead iNGO for assistance in procuring and distributing contraceptive commodities and supplies procured by UNFPA.

### Step 3: Assess the environment (i.e. the conditions within which the scale-up occurs)

The DRC government began to signal its support of family planning around 2012, initially with a focus on reducing maternal mortality. In 2013, it publicly committed support for family planning at the 2013 International Conference on Family Planning in Addis Ababa. In 2014, the Minister of Health released a National Multisectoral Strategic Plan for Family Planning: 2014–20, which called for increasing modern contraceptive prevalence from 6.5% to 19% among all women of reproductive age (Mukaba *et al.*, 2015). In 2015, the Secretary General of the MOH issued a letter that endorsed the testing of this new approach to contraceptive service delivery; based on monthly briefings, he provided continued guidance for the implementation of the scale-up process. Another enabling factor was the support from donors, who viewed the potential of this intervention to transform the system for long-term sustainability*.* However, the scale-up initiative faced multiple challenges: the sheer number of nursing schools in the country (477), the vast expanse of the country, weak communication infrastructure and the lack of financial and human resources for nursing training, to name a few.

### Step 4: Enlist the commitment of individuals and organizations that will promote and facilitate wider use of the innovation

In this case, ‘wider use’ referred to scale-up within the D6 nursing school system. However, the resource team consisted of multiple organizations that contributed to this process. A local NGO specializing in family planning training and PNSR personnel conducted training of master trainers (among D6 personnel and Chief Medical Officers of health zones), who in turn trained the nursing school instructors in preparation to train the nursing students. One instructor per school was selected as ‘focal point’, based on expressed interest, frequent interaction with the students and (in many cases) experience in serving as an advisor to them. His/her role was to supervise nursing students during the community-based service provision. Later during the extension of the innovation to other provinces, the local NGO specialized in family planning training and PNSR personnel trained pools of Master trainers from each province, who in turn trained instructors as new schools were incorporated into the scale-up.

The D5, responsible for the SNIS, worked with the D6 and the lead iNGO personnel to include data from the nursing students as a separate entry field in the SNIS. The system attributed the nursing students’ output to the health zone in which they were working, separate from that generated by fixed facilities and other community-based programmes in each health zone. This change to the SNIS institutionalized a mechanism of tracking results generated by the nursing students.

### Step 5: Institutionalization of the innovation: clarifying how the policy changes will be initiated and how they will be funded

The D6 team sought to implement the policy change outlined in the National FP Strategic Plan, requesting a revision of the family planning curriculum of nursing [and medical] students throughout the country, recognizing that the expanded adoption/application of the new community-based service delivery module would need to occur in staggered fashion for both logistical and financial reasons. This process also aligned with the task-sharing policy on human resources for health as well as the ongoing reform on competency-based training approaches for training institutions.

**Figure 1:**
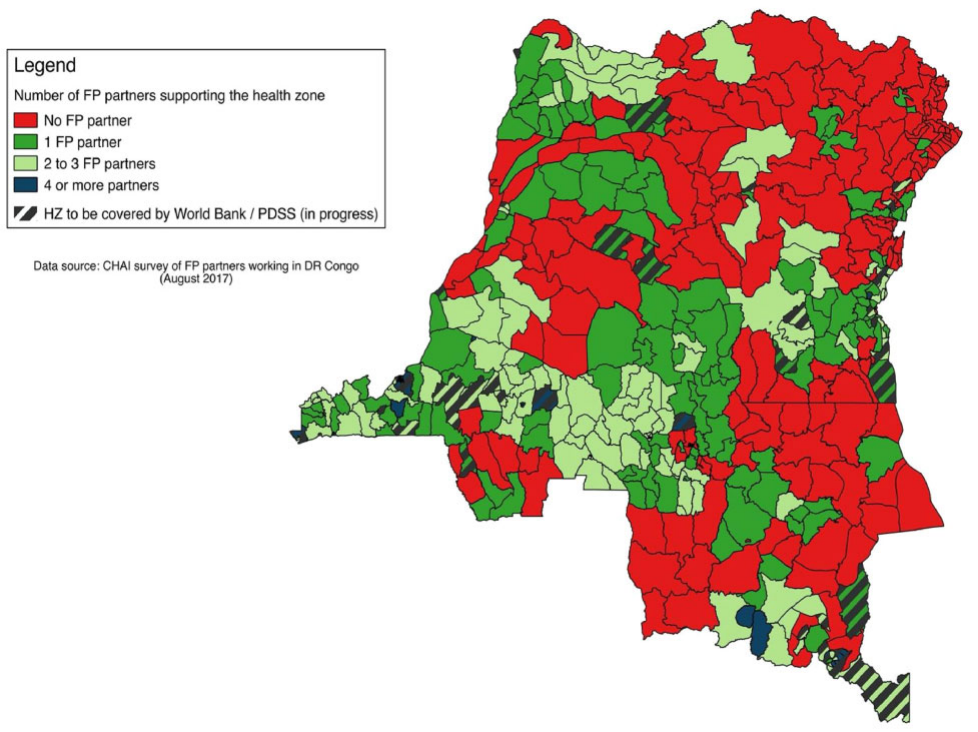
Number of partner organizations supporting family planning in the 519 health zones of the DRC, as of 2017 (source: CHAI survey).

Family planning in the DRC is highly donor-dependent (Kwete *et al.*, 2018), although this scale-up process involved a network of schools that existed independent of external funding. Donor funding for family planning does not cover all 519 health zones, although numerous health zones benefit from the support of more than one donor. The most recent map of donor funding, produced by the Clinton Health Access Initiative (CHAI) in 2017, reflects this complex funding situation (see Figure 1).

Three donors contributed substantially to this initiative. From 2015 to 2018, the Bill and Melinda Gates Foundation (BMGF) supported a series of pilot studies that demonstrated the feasibility and acceptability of using nursing students as community-based distributors of contraceptives (Bertrand *et al.*, 2018; Hernandez *et al.*, 2020). A private philanthropist interested in family planning in the DRC provided a grant to expand the nursing school model within Kinshasa and replicate it in Kongo Central between 2016 and 2018. In 2019, the BMGF co-financed this initiative, which by then included a third province, Lualaba. In late 2019, FONAREDD funded PROMIS-PF, a new 3-year project to scale-up family planning in 11 provinces. This new funding stream allowed for expansion of the nursing student model to four additional provinces: North Kivu, South Kivu, Tshopo and Haut Katanga.

TILLC provided the D6 with small incentives (‘transport’) for focal points, students and health zone officials to participate in service delivery, as well as arranging for them to have the commodities (contraceptives and supplies) needed for service delivery.

The MOH through the D6 made important cost-sharing contributions. Whereas the first cohort of nursing students and their instructors benefitted from perks during the feasibility study (e.g. refreshments during the training, transport fees), the D6 aligned the implementation of the module with the ‘bare-boned approach’ of nursing school operations. Specifically, the D6 schools absorbed costs, such as providing classrooms instead of hiring special venues for the training and paying instructor salaries as part of their normal teaching load (although supervisors received a small bonus for supervising the community-based practicums). This contribution reduced the full cost of family planning training by 30%.

To enhance the sustainability of this initiative, the D6 sought support from ANAPECO, the national parent–teacher association. Through national and provincial level meetings with these parents’ groups, it was possible to explain to parents the benefit of having their sons and daughters trained in contraceptive service delivery using a competency-based model. ANAPECO responded by requesting that all parents pay a slight increase in tuition over the 4 years of training, although the students only begin the family planning module in Year 3. The contribution of ANAPECO reduced the cost of training nursing students by an additional 20%, decreasing donor dependence.

### Step 6: Horizontal scale-up: prepare for the adoption of the innovation at scale

**Figure 2:**
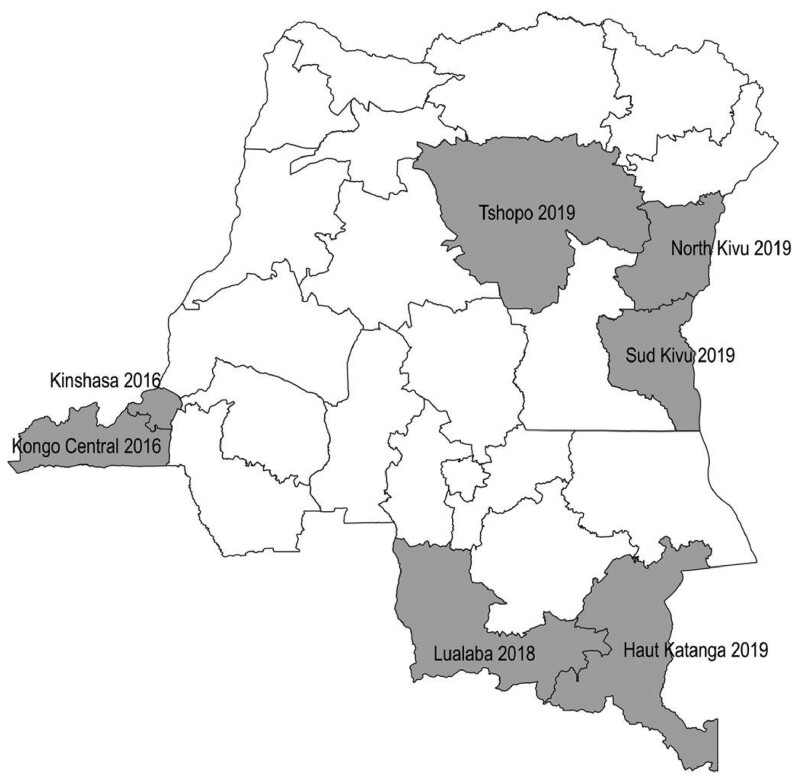
Provinces in which the community-based family planning services module was incorporated into the curriculum of third-year nursing students in the DRC, with start date.

In the case of the DRC, the financial and human resources were insufficient to go to scale nationwide in the short-run. Rather, the curriculum was incorporated in new provinces as financial support became available. Figure 2 shows the pace at which the training was introduced in new provinces and new schools over time, which tracked closely with the availability of funding (described in Step 5). Although the MOH is theoretically responsible for providing primary health care, including family planning, through the 26 provinces of the DRC, it falls far short of achieving this goal. Family planning (like other major health areas in the DRC) is highly dependent on donor funding. In this case, the funding from three donors (cited above) supported both training costs and service delivery. The interest to the donors is that the nursing school model represents another vehicle for increasing access to contraception in partnership with a government entity that will continue beyond the lifetime of a specific donor-funded project. The current donor support for this model runs through 2022. In addition, USAID through the Elizabeth Glazer Pediatric AIDS Foundation (EGPAF) has joined the scale-up process and will support five nursing schools to incorporate family planning into the curriculum.

### Step 7: Scaling up the innovation through diversification, i.e. testing the delivery of other types of services provided by nursing students

Based on the promise of the nursing student model, in 2017, the BMGF funded a new 3-year project MOMENTUM, designed to test the effectiveness of using nursing students in Kinshasa to provide an expanded package of community-based services targeting first-time mothers age 15–24 years old and their male partners. The project seeks to improve care-seeking and maternal and neonatal health household practices, increase post-partum contraceptive use and improve gender-equitable attitudes and beliefs among male partners. ‘Diversification’ refers to the fact that the same user-organization(the D6) undertook the training of the same cadre of personnel (nursing students) on an expanded set of topics (best practices for prenatal, care-seeking and maternal and neonatal health household practices, as well as gender-equitable attitudes and beliefs among male partners). Also, the mode of service delivery—in particular home visits to pregnant women—differed from the community-based outreach approach used in the previous pilots. The results of an impact evaluation of the intervention, based on a pre-test post-test design with comparison group, will be forthcoming in late 2021. These findings will be used as a basis for identifying aspects of the MOMENTUM intervention that may be institutionalized within the nursing schools’ curriculum.

Also, the previously cited pilot studies resulted in additional innovations in service delivery as of 2019: using fourth year nursing students to remove Implanon NXT and to train interested clients in DMPA-SC self-injection; and to include emergency contraception in the range of methods delivered.

### Step 8: Addressing spontaneous adoption/expansion of the innovation

The D6 controls the selection of schools to receive the module at the national level. Thus, any spontaneous replication of the new curriculum outside the government-accredited nursing school network is unlikely.

### Step 9: Finalizing the scaling-up strategy and identifying next steps

There has been strong political pressure to move quickly and, as it typically happens, the management of scale-up (institutionalization and expansion) remains an iterative process of implementation, observation, reflection and adaptation, using the ExpandNet/WHO guidance. Provinces not yet part in the scale-up are pressuring the D6 to be included, but funding remains a constraint. An important next step is to work with government, donors and other stakeholders to develop a mechanism to ensure sufficient contraceptive commodities, possibly through support from UNFPA. The D6 plans to develop guidelines that formalize arrangements with potential new partners interested in using nursing students in their own service delivery activities.

## Materials and methods

The research designed around the scale-up process reflected the dual objective of strengthening the capacity of DRC nursing schools to provide competency-based training in family planning, while expanding access to contraceptive services delivered at the community level. Multiple data sources were used to generate evidence on contraceptive uptake, perceptions from D6 staff involved in implementation and the students’ use of the training post-graduation.

### Measuring increased access and contraceptive uptake

In this article, access refers to the number of provinces and health zones within provinces in which nursing students provide contraceptive counselling and services.

In terms of uptake, the most widely used output indicator of performance in international family planning programmes is couple-years of projection (CYP), calculated on the basis of the volume of each type of contraceptive distributed to clients in a given programme, then converted to a measure of the estimated protection provided to a woman over a 1-year period (USAID, 2019). This indicator is derived from routine services statistics.

The DRC has made substantial strides in recent years to develop its SNIS for collecting and reporting service statistics across the health sector, using the DHIS2 platform. However, the system was not functional until 2019. Thus, in the early years of the scale-up process (2015–18), we relied on routine statistics reported by the students to their school’s focal point, who then submitted the data directly to the project team.

As of January 2019, TILLC started reinforcing the use of the SNIS to make it the primary data source for the volume of each contraceptive method distributed by the students and by health zone during outreach events or other community-level service delivery for the three long-standing provinces: Kinshasa, Kongo Central and Lualaba. Because the SNIS still lagged behind in integrating nursing school service statistics from the four newer provinces, we used services statistics directly transmitted by the schools to increase accuracy of reporting. Although progress has been made in the number of nursing schools reporting through the SNIS, continuous access to the internet remains an obstacle.

### Eliciting the perspectives of key nursing school personnel using key informant interviews

In January to April 2019, a 14-person team from the implementing organizations (D6, the local NGO specialized in training, PNSR and the lead iNGO) carried out qualitative data collection to elicit the perspectives of those involved in integrating the new competency-based family planning training module into the existing curriculum. Rather than a formal research study, it was conducted as a programmatic exercise intended to build capacity among the D6 personnel and foster local ownership, while also identifying areas for future improvement. The authors recognize that the use of data collectors who were familiar with the process and some of the key actors could have resulted in a courtesy bias in responses. However, the informal atmosphere of a group meeting was intended to put respondents at ease and increase willingness to speak as compared with a more structured interview with an unknown interviewer.

From 93 schools that had integrated the module during the trial period (September 2017 to July 2018, and September 2018 to July 2019), a convenience sample of 30 was selected: all 22 schools (Kinshasa, 16; Kongo Central, 6) from the first cohort and eight randomly selected schools (4 per province) from the second cohort. Two member teams simultaneously visited each school for a 1- to 2-day period in late March 2019. In each school, they interviewed two to four informants in French, including the principal, focal point and other instructor(s) who had previously participated in a 2-week training-of-trainers organized by D6 at the provincial level. They used a previously developed discussion guide that covered two main points: perceptions of the value of scale-up this type of family planning training in their schools, and suggested changes in the process for the expansion to other schools. One of the two-person team took notes during the interviews, which lasted for 30 min or less and were scheduled to avoid disrupting classes. Members of the full team met in late April 2019 to synthesize the key results. Each team reported its findings on the two main topics, which were written up as a set of notes. The lead author systemically reviewed these notes, synthesized the main ideas and drew conclusions on key topics for the exercise. (These sessions were not transcribed, nor was software used in this analysis.)

### Conducting a post-graduation phone survey among D6 graduates

From June to August 2019, a separate team conducted a phone survey (quantitative) among nursing school graduates to assess their career path since graduation, including opportunity to provide contraceptive services. The researchers worked with D6 personnel to obtain names and contact information from students in six schools in Kinshasa and five schools in Kongo Central trained in family planning between 2015 and 2018. Interviewers experienced with phone surveys were recruited and trained for 2 days, then conducted three practice interviews, before starting the data collection. (A few minor changes in wording were made after these practice sessions.) A supervisor, two interviewers in Kinshasa and one interviewer in Kongo Central attempted to reach all graduates and interview them by phone. If unreachable, the D6 officials contacted them through friends and requested that they come to the school to be interviewed. The interviews ran from 15 to 25 min and were conducted in French, Lingala (local language in Kinshasa) or Kikongo (local language in Kongo Central). The interviewers entered the respondents’ answers directly into a smartphone, programmed with an ODK app. The supervisor double checked data entry before submission in the server. After quality control and consistency checks, data were exported into SPSS 23.0 and Stata 15 for analysis.

Descriptive statistics were used to describe the basic characteristics of the study data. Means and standard deviations (SDs) were calculated for normally distributed continuous variables, while proportions with their 95% confidence intervals (CIs) were calculated for categorical variables. We used the Z-test to compare the proportion between two groups, *t*-test to compare the mean between two groups and Pearson’s chi-square test to test for associations. The logistic regression allowed us to identify factors associated with use of the family planning training on the job and to obtain adjusted odds ratio (aOR) and 95% CIs. Variance-inflation factors were estimated to assess multicollinearity. A significance threshold of α = 0.05 was used for all tests.

## Results

### Increased access and contraceptive uptake

As of November 2019, the family planning curriculum had been incorporated into the nursing school training in 147 nursing schools across 61 Health Zones in seven provinces (see Figure 1). This number represents 28.1% of the all nursing schools in the country, with services provided in 17.7% of the health zones in the country.

**Figure 3:**
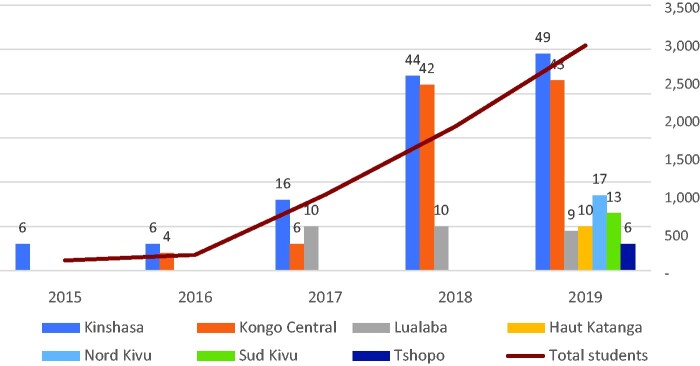
Number of schools in which the new family planning module was incorporated, by year and by province (note: the red bar denotes the total number of schools per year that incorporated the training).

Figure 3 shows the progressive integration of the family planning module at the provincial level (by number of schools) between September 2017 and November 2019. A total of 6083 nursing students benefitted from this training.

Through outreach and campaign events, the nursing students in these schools generated a total of 461 769 CYP between 2016 and 2019. Table 1 details the volume of CYP produced each year in the participating provinces.

**Table 1:** Total volume of CYP generated by students per year and per province

Province	2016	2017	2018	2019	Total CYP 2016–2019
Kinshasa	327	33 196	97 474	186 833	317 829
Kongo Central	9	4659	49 996	52 846	107 510
Lualaba		1273	3728	9547	14 547
Ht Katanga				6302	6302
Nord Kivu				8862	8862
Sud Kivu				4063	4063
Tshopo				2655	2655
** Total**	**336**	**39 127**	**151 198**	**271 108**	**461 769**

**Figure 4:**
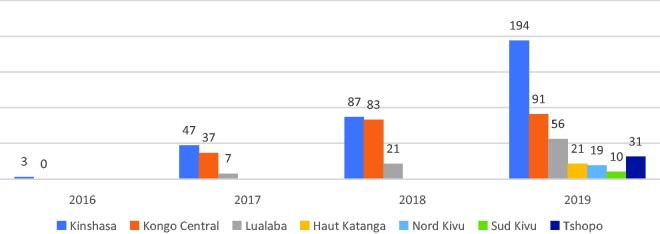
Mean CYP provided per trained student and per year for each province.

Figure 4 indicates the mean CYP provided per trained student and per year for each province. The mean CYP per student increased gradually over time, with by far the highest level in Kinshasa (194), followed by Kongo Central (91) and Lualaba (56) by 2019. This ranking reflects the chronological order in which the provinces incorporated the FP training; it may reflect the population density of the cities served by the nursing schools. By contrast, the number of CYP per student is relatively low in Tshopo (31), Haut Katanga (21), Nord Kivu (19) and Sud Kivu (10), because the training only occurred in the final 2 months of 2019; in addition, shortages of some contraceptives reduced uptake.

Overall, the volume of CYP per student and per year increased from 5 CYP in 2016 (with two provinces involved) to 60 CYP per student in 2019 (with seven provinces involved); data not shown.

### Perceptions of key D6 personnel regarding the scale-up process

A total of 77 key actors were interviewed: 29 principals, 30 instructors and 18 focal points (from the first cohort only); 5 others were recruited but were not able to participate in the full session and were excluded from the analysis. This sample included 52 key informants from Kinshasa, 25 from Kongo Central. Although each participant was interviewed separately, the responses were very similar across all the three categories of personnel, suggesting that schools had followed the recommendation to work as a team in introducing the training approach.

Key findings were as follows. The personnel in Kongo Central were uniformly satisfied with the integration of the training approach into the curriculum. By contrast, satisfaction was slightly lower (approximately four in five) in Kinshasa province, due to difficulties (including multiple postponements) experienced in implementing the planned training session in 2018–19. Nevertheless, all three categories of personnel in both provinces favoured the integration of this new module *on a lasting basis*, because of its undisputable value-added for the schools, the students and the entire community.

The large majority were favourable towards the incorporation of the new module into the curriculum and felt it could be sustained within the routine operation of the nursing school system. By contrast, they experienced more difficulties in implementing the service delivery component (contraceptive distribution at the community level) for lack of the materials needed to do so: a constant stock of the full range of contraceptive methods, supplies needed for the administration of certain methods (e.g. cotton, antiseptic) and inventory cards. The designated focal points for this initiative cited the heavy additional workload that these interventions entailed, not only for them but also for the nursing school system more generally.

Using a highly participatory approach, the respondents identified a few priority mid-course corrective measures (e.g. guidelines for supervision of nursing students, strengthening the coordination with health zones/health centre staff, data management of service statistics). These changes were incorporated into the annual planning for all the 2019–20 cohort of 147 nursing schools across seven provinces.

### Follow-up study among D6 nursing graduates on their experience in the programme and post-graduation

The list of graduates obtained from nursing school officials included 295 from Kinshasa and 59 from Kongo Central. Of these, we were able to reach and interview 223 from Kinshasa (75.6%) and 49 graduates from Kongo Central (83.1%), for a combined response rate of 76.8%.

In both provinces, three-quarters of the graduates were female (73.1%, Kinshasa; 85.7% in Kongo Central); Table 2. The mean age was 25.4 years old, although graduates in Kinshasa were far younger (24.4 years) than those in Kongo Central (31.7 years). Relatively few in either province were married or living in union, though the percentage was higher in Kongo Central (32.6%) than in Kinshasa (10.3%). Given differences in age and marital status, it is not surprising that graduates in Kongo Central were more likely to have children (69.4%) than those in Kinshasa (17.5%).

**Table 2:** Sociodemographic characteristics of the nursing graduates Total

	Overall		Kinshasa		Kongo central	
	*n*	%	*n*	%	*n*	%
Age (mean ±SD)	25.4 ± 7.5		24.0 ± 6.4		31.7 ± 9.0***	
Age (years)				***		
<25 ans	183	67.3	176	78.9	7	14.3
25–29 ans	43	15.8	25	11.2	18	36.7
≥ 30 ans	46	16.9	22	9.9	24	49.0
Sex						
Male	67	24.6	60	26.9	7	14.3
Female	205	75.4	163	73.1	42	85.7
Marital status				***		
Never married	228	83.8	197	88.3	31	63.3
widowed/divorced	5	1.8	3	1.3	2	4.1
Married/in union	39	14.3	23	10.3	16	32.7
Living children				***		
No	199	73.2	184	82.5	15	30.6
Yes	73	26.8	39	17.5	34	69.4
Living children				*		
1–3	58	79.5	27	69.2	31	91.2
4+	15	20.5	12	30.8	3	8.8
73	100.0	39	100.0	34	100.0	

SD, standard deviation; *P < 0.05; **P < 0.01; ***P < 0.001.

Regarding their training experience, close to 90% had graduated in 2017–18 (reflecting the far larger numbers trained in those 2 years than in 2015–16); Table 3. Nine in 10 graduates confirmed that they had been trained in contraceptive methods. Nine in 10 reported to have participated in a campaign event (community-level service delivery) and to have provided Implanon NXT (which sets them apart from community-health workers who are not authorized to administer this method). Just over half (54.7% in Kinshasa, 63.3% in Kongo Central) had ever taught a client to self-inject with DMPA-SC (still considered an innovative practice in the DRC).

**Table 3:** Experience with family planning training in nursing school

	Overall		Kinshasa		Kongo Central	
	*N*	%	*N*	%	*N*	%
Year graduation				^***^		
2015–16	26	9.6	25	11.2	1	2.0
2017	104	38.2	72	32.3	32	65.3
2018	142	52.2	126	56.5	16	32.7
Received training on contraceptive meth				^*^		
No	18	6.6	18	8.1	0	0.0
Yes	254	93.4	205	91.9	49	100.0
Participated in campaign event				^*^		
No	20	7.4	20	9.0	0	0.0
Yes	252	92.6	203	91.0	49	100.0
Taught self-injection						
No	119	43.7	101	45.3	18	36.7
Yes	153	56.3	122	54.7	31	63.3
Provided Implanon NXT				^**^		
No	29	10.7	29	13.0	0	0.0
Yes	243	89.3	194	87.0	49	100.0
Satisfaction with FP training at School^a^				^**^		
Completely satisfied	191	75.2	146	71.2	45	91.8
Somewhat satisfied	63	24.8	59	28.8	4	8.2
Usefulness of FP training^a^				^*^		
Completely useful	221	87.0	173	84.4	48	98.0
Somewhat useful	33	13.0	32	15.6	1	2.0
Total	272	100.0	223	100.0	49	100.0

* *P* < 0.05;

** *P* < 0.01;

*** *P* < 0.001.

a Among those who training on contraceptive methods.

The majority (75.2%) of graduates reported to be completely satisfied with their FP training; the percentage was higher in Kongo Central (91.8%) than in Kinshasa (71.2%); Table 3. Similarly, the Kongo Central graduates were more likely to consider the training to have been useful (98.0%) than did Kinshasa graduates (84.4%).

A primary aim of the study was to assess the extent to which the graduates of this family planning training had been able to put it to use post-graduation. Consistent with the high levels of unemployment in the DRC, less than half the nursing graduates (40.4%) were formally employed, with employment higher in Kongo Central (59.2%) than Kinshasa (36.3%); Table 4. Among the employed, the majority worked in a public or private health facility; and 9 in 10 (87.7%, Kinshasa; 100%, Kongo Central) had had opportunity to provide FP services on the job.

**Table 4:** Employment status and use of family planning training post-graduation

	Overall		Kinshasa		Kongo Central	
	*n*	%	*n*	%	*n*	%
Currently employed				^**^		
No	162	59.6	142	63.7	20	40.8
Yes	110	40.4	81	36.3	29	59.2
Place of employment (*n* = 110)^a^						
Public hospital/health centre	31	28.2	20	24.7	11	38.0
Private hospital/health centre	58	52.7	47	58.0	11	38.0
Pharmacy	14	12.7	9	11.1	5	17.2
Others	7	6.4	5	6.2	2	6.8
Opportunity to provide FP in current job (*n* = 110)^a^				^*^		
No	10	9.1	10	12.3	0	0.0
Yes	100	90.9	71	87.7	29	100.0
Type of FP work (*n* = 100)^b^						
Counselling	99	99.0	70	98.6	29	100.0
Contraceptive provision	81	81.0	58	81.7	23	79.3
Referral	39	39.0	36	50.7	3	10.3
Other	1	1.0	1	1.4	0	0.0
Training helpful in current work				^*^		
No	29	10.7	28	12.6	1	2.0
UYes	230	84.6	182	81.6	48	98.0
Don’t know	13	4.8	13	5.8	0	0.0
Ever provided FP services as volunteer				^**^		
No	62	23.8	59	27.8	3	6.2
Yes	198	76.2	153	72.2	45	93.8
Ever provided services at National FP day				^***^		
No	194	71.3	147	65.9	47	95.9
Yes	78	28.7	76	34.1	2	4.1
Ever provided services at campaign (Lelo PF)				^***^		
No	150	55.1	111	49.8	39	79.6
Yes	122	44.9	112	50.2	10	20.4
Total	272	100.0	223	100.0	49	100.0

* *P* < 0.05;

** *P* < 0.01;

*** *P* < 0.001.

a Among those employed.

b More than one response possible and percentage is calculated for those who got Opportunity to provide FP in current job.

We asked all respondents (employed or not) if they had ever provided FP services as a volunteer: three-quarters in both provinces had. Far fewer—approximately one in three—had provided services at a National Family Planning Day. Graduates in Kinshasa (50.2%) were more likely to have participated in a *Lelo PF* community outreach campaign than those in Kongo Central (20.4%), again reflecting the greater frequency of this type of outreach in Kinshasa. However, given the phrasing of these three questions, we cannot be certain if the graduates completed these volunteer activities during or after their training.

Ideally, students trained in this programme would be able to find employment that allowed them to put their training to use in providing FP services. A total of 110 graduates (40.4% of those trained; both provinces combined) were thus employed. Table 5 presents an analysis of the factors associated with this outcome. Of the five variables tested, three showed a clear association. Graduates over age 30 were six times more likely than those under 25 to have this type of employment (aOR 5.61; 95% CIs 1.68–18.76). Although males represented only one-third of graduates, they were over twice as likely as females to have a job where they used their training (aOR 1.92; 95% CIs 1.04–3.56). Graduates from Kongo Central were also more likely than those in Kinshasa to have a job that used their family planning training (aOR 2.70; 95% CIs 1.22–6.00).

**Table 5:** Factors associated with use of the family planning training on the job

	Use of the FP training on the Job^a^								
		No		Yes					
	*N*	*n*	%	*n*	%	crude OR	95% CI	Adjust OR	95% CI
Age (years)									
<25	71	48	67.6	23	32.4	1		1	
25–29	141	91	64.5	50	35.5	1.15	0.63–2.09	1.03	0.53–1.99
≥30	42	15	35.7	27	64.3	3.76	1.68–8.39	5.61	1.68–18.76
Sex									
Male	66	35	53.0	31	47.0	1.53	0.87–2.69	1.92	1.04–3.56
Female	188	119	63.3	69	36.7	1		1	
Marital status									
Not married	218	135	61.9	83	38.1	1		1	
Married/in union	36	19	52.8	17	47.2	1.46	0.72–2.96	0.58	0.20–1.72
Living children									
No	183	117	63.9	66	36.1	1		1	
Yes	71	37	52.1	34	47.9	1.63	0.93–2.84	0.58	0.23–1.45
Province									
Kinshasa	205	134	65.4	71	34.6	1		1	
Kongo central	49	20	40.8	29	59.2	2.74	1.45–5.18	2.70	1.22–6.00
Year graduation									
2015–16	25	13	52.0	12	48.0	1		1	
2017	89	50	56.2	39	43.8	0.85	0.34–2.06	0.53	0.20–1.40
2018	140	91	65.0	49	35.0	0.58	0.25–1.38	0.52	0.21–1.33
Total	254	154	60.6	100	39.4				

a This table is based on those respondents who reported to have received training in contraceptive methods.

## Discussion

This article describes the process and results of a 4-year initiative to institutionalize family planning into the third year of the curriculum of nursing schools throughout the DRC. The innovative component is the use of these nursing students in contraceptive counselling and service provision at the community level during their training, as a means of increasing contraceptive access. This case study is of particular interest, because it demonstrates that progress is possible even in the highly challenging environment of the DRC. This country ranks 176 of 189 countries and territories on the Human Development Index (2018) and has experienced chronic political conflict, especially in the eastern part of the country, for decades. Moreover, the topic of family planning remains somewhat controversial in this country, given the high fertility norms and low status of women (Kwete *et al.*, 2018).

The research conducted to inform this scale-up yielded key findings. The scale-up was effective in increasing access to modern contraceptive methods, as measured by the volume of CYP generated. The highest levels of CYP corresponded to longevity of the programme and population density of the communities served. Personnel in the D6 responsible for integrating family planning into the nursing curriculum recommended scale-up, despite the challenges of implementing the service delivery component, because it strengthened the training offered and benefitted the community. The follow-up survey among nursing graduates of the programme reflected the high levels of unemployment in the DRC; only 4 in 10 graduates had found paid employment. Yet among those employed, 9 in 10 had had the opportunity to provide family planning services on the job. This finding underscores the importance of the programme, not only for service delivery while the nursing students are still in training, but the potential longer-term impact on the healthcare labour force.

Despite a promising start, the scale-up process met with multiple challenges. The first related to obtaining a sufficient supply of contraceptive commodities to fulfil the needs generated by the scale-up. The problem is not unique to this initiative; existing donor and government funding is insufficient to meet the growing demand for a range of contraceptives in the DRC. The nursing students in the four provinces incorporated in late 2019 received training and conducted several practice sessions at the community level, but the lack of contraceptives stymied their ability to launch community services post-training. The logistical challenges are further magnified by the large number of nursing schools and the vast expanse of the country (the size of western Europe).

A second challenge was to ensure quality of the training and service delivery components, as the process was scaled up beyond the initial sites in Kinshasa to provinces over a thousand miles away. Although a core team was responsible for the training across provinces, it is hard to manage and supervise the activities in multiple provinces. Further complications include the weak health system, unreliable communication networks and very limited resources. These problems characterize the public health infrastructure in the DRC, within which this new model will need to operate. One means of addressing this problem has been to link the D6 service delivery to the programme of the local health zones.

A third challenge is the average age of the nursing students: 15–18 years old. At one point, a rumour circulated that minors were involved in the distribution of contraceptives, which would have caused problems for the MOH. Yet by the time the students reach the third year of their nursing training, most are 18. At the same time, their age can be a benefit, since prospective adolescent clients may find them more approachable than older providers.

As one looks to the future, the major challenge will be financial. Whereas the training component has strong potential for sustainability, now that many instructors have themselves been trained and have the experience of training new cohorts of students, the service delivery component requires a continuous influx of cash. Donors have funded this model (in addition to others) because of its promise to increase contraceptive access in the short-run and develop the next generation of health workers training in family planning in the long run. However, it is unlikely that the D6 could continue to operate the community-level service delivery without external funding.

The DRC experience contributes to the growing literature on the scale-up of health interventions in low- and middle-income countries. Seven articles are particularly relevant because they used the ExpandNet/WHO framework and/or addressed reproductive health intervention in sub-Saharan African countries (with two exceptions, Bangladesh and Vietnam). In contrast to the DRC where the ExpandNet/WHO framework was adopted from the start, most others have used it as a retrospective evaluation tool (Dickson *et al.*, 2011; Hainsworth *et al.*, 2014; Huaynoca *et al.*, 2014; Hobday *et al.*, 2019). There are multiple similarities in the findings from the DRC and those from other countries.

*Strong national level governmental support* is essential across intervention scale-ups, especially support for policy changes that create an environment conducive for scale-up success. For example, the governments of Mozambique and Nigeria previously made public commitments to support the health topic that the scale-up addressed and passed policy to enable a national-level expansion (Dickson *et al.*, 2011; Callaghan-Koru *et al.*, 2019; Hobday *et al.*, 2019).

*Coordination and support among stakeholders at all levels* is vitally important. In the initial phase of the process, engaging and uniting stakeholders was a common foundation on which to build (Dickson *et al.*, 2011; Ghiron *et al.*, 2014; Hainsworth *et al.*, 2014; Huaynoca *et al.*, 2014; Hobday *et al.*, 2019). In Mozambique, multisectoral coordination was cited as a key element in the scale-up of adolescent-friendly contraceptive services (AFCS), yet in the same country a breakdown in coordination between different levels of government, the MOH, NGOs and donor organizations led to difficulties in implementing the scale-up of community distribution of misoprostol for post-partum haemorrhage (Hainsworth *et al.*, 2014; Hobday *et al.*, 2019). Reaching out to all stakeholders, including those that could oppose the project, was also important. In Nigeria, the national scale-up of a Comprehensive Sex Education curriculum included the creation of Advisory and Advocacy Committees, consisting of key stakeholders such as traditional and religious leaders, school administrators and representatives of teachers’ unions and parents associations (Huaynoca *et al.*, 2014). By contrast, the exclusion of important stakeholders in some projects stymied full implementation and reduced the likelihood of achieving sustainability (Huaynoca *et al.*, 2014). A PHE project in Uganda and Kenya found that while the inclusion of stakeholders led to access to resources that could improve sustainability, some stakeholders strongly advocated for expansion before there was evidence that the project was succeeding (Ghiron *et al.*, 2014).

*Scale-up is often donor-dependent*, as was the case in Mozambique, Ghana, Ethiopia, Tanzania, Nigeria and Vietnam, with limited financial contribution from the government (Hainsworth *et al.*, 2014; Hobday *et al.*, 2019). During the scale-up of comprehensive sex education in Nigeria, both the federal and state governments provided funding for implementation; however, shortages in funding led to uneven national implementation as resources were inadequate for the printing and dissemination of materials (Huaynoca *et al.*, 2014).

*Scale-up often uses a phased approach*. Horizontal expansion in the DRC resulted from logistical and financial constraints. Elsewhere, countries adopted the phased approach to allow lessons from previous phases to improve implementation in later phases (Hainsworth *et al.*, 2014; Hobday *et al.*, 2019).

*Vertical scale-up* consisted of both policy change and the integration of the innovation into the existing system and government workplans and budgets at all levels (Hainsworth *et al.*, 2014; Huaynoca *et al.*, 2014; Callaghan-Koru *et al.*, 2019; Hobday *et al.*, 2019). While the majority of scale-ups enacted both horizontal expansion and vertical institutionalization, often simultaneously, the incorporation of diversification was less common. In the DRC, new funding led to the creation of the MOMENTUM project (which used the same cadre of nursing students to provide counselling and post-partum family planning service delivery, in addition to other services for maternal and neonatal health, by conducting home visits to first-time mothers). Similarly, in Mozambique, new donors supported the inclusion of HIV care and treatment in the national-level scale-up of AFCS (Hainsworth *et al.*, 2014).

*Increasingly, scale-up relies on the national health information system for monitoring purposes*. Scale-ups in Ghana, Mozambique, Tanzania, Vietnam and Bangladesh used health management information systems to track monitoring data (Hainsworth *et al.*, 2014; Callaghan-Koru *et al.*, 2019). However, some scale-ups found HMIS data to be insufficient for project purposes (as did the DRC in some provinces) and supplemented it with other data sources, and a few created monitoring systems outside of the HMIS (Huaynoca *et al.*, 2014; Keyonzo *et al.*, 2015; Callaghan-Koru *et al.*, 2019).

The current analysis has several limitations. First, it is based on a single case, which may be particular to the political and public health context of the DRC, with limited generalizability. Second, the findings on the perceptions of D6 personnel disproportionately reflect the experience of schools that participated in the early phase of scale-up, rather than the larger cohorts that joined in 2019. Third, the follow-up survey of nursing graduates included students who were trained before the improved curriculum was introduced in 2017. Also, the questions regarding volunteer work in family planning service delivery did not distinguish between work during nursing school and work since graduation.

In conclusion, the case study provides promising results for the scale-up of an innovative strategy for both increasing access to contraception in the short-run and strengthening the capacity of future healthcare workers in family planning service delivery in the long run. It reflects the value of using a tested framework (ExpandNet) to navigate the steps in the scale-up process. The model has strong country buy-in from the D6 leadership, which plans to expand the use of competency-based training more widely in its curriculum. Although continued scale-up of the model will require financing far beyond what the MOH could provide, donors look favourably on investments that have a lasting impact on systems, such as the nursing curriculum for the country. Future research to guide the process should include ongoing assessment of the policy environment for the scale-up, ability of the D6 to maintain fidelity to the model with the expansion to new provinces, quality of services provided by students in the programme and cost-effectiveness of the nursing student model of service delivery compared with other forms of delivery.

## Footnotes

1 The names of the Directorates in French are: D5 = Direction des soins de santé primaire, integrated in DGOGSS; D6= Direction de l’Enseignement des Sciences de Santé; and D10 = Direction de la famille et des groupes spécifiques.

2 The family planning curriculum has been introduced into the training for nurses and midwives, but for simplicity, we cite only the nursing training in this article.

3 In 2019, the D5 was subsumed into the DGOGSS (Direction Générale d'Organisation et de Gestion des services de santé, or Directorate for the Organization and Management of Health Services). For simplicity, we have retained the D5 designation in this article.
